# Clinical and aesthetic outcomes of transumbilical single-port versus multi-port laparoscopic ovarian cystectomy

**DOI:** 10.3389/fendo.2026.1856062

**Published:** 2026-06-30

**Authors:** Qingquan Shi, Yunan He, Jiahui Qiu, Jinhong Li

**Affiliations:** 1 Center of Reproductive Medicine, West China Second University Hospital, Sichuan University, Chengdu, China; 2 Key Laboratory of Birth Defects and Related Diseases of Women and Children, Ministry of Education, Chengdu, China

**Keywords:** aesthetic satisfaction, clinical outcomes, inflammatory response, ovarian cystectomy, ovarian reserve, single-port laparoscopy

## Abstract

**Introduction:**

Ovarian cysts often require surgery. This study compared clinical outcomes and aesthetic satisfaction between transumbilical single-port (SP-LC)s and traditional multi-port laparoscopic cystectomy (MPL).

**Methods:**

A retrospective analysis was conducted on patients undergoing laparoscopic ovarian cystectomy from October 2023 to September 2025. Patients were divided into MPL and SP-LC groups based on surgical technique. Perioperative parameters were recorded. Postoperative pain was assessed using the Visual Analog Scale (VAS) at 24 and 72 hours. Inflammatory markers were measured preoperatively and 24 hours postoperatively. Ovarian reserve function was evaluated preoperatively and at 1 and 3 months postoperatively. Postoperative complications were documented. Aesthetic satisfaction was assessed at discharge, and scar quality was evaluated using the Patient Scar Assessment Scale (PSAS)ss at 1 and 3 months postoperatively.

**Results:**

SP-LC showed better perioperative recovery than MPL. It was associated with less intraoperative blood loss (43.06 ± 11.79 vs. 48.12 ± 12.85 mL, P = 0.014), earlier first exhaust (15.82 ± 5.21 vs. 18.47 ± 5.74 h, P = 0.004), earlier ambulation (13.89 ± 3.17 vs. 15.63 ± 3.51 h, P = 0.002), and shorter hospital stay (3.94 ± 0.85 vs. 4.31 ± 0.92 d, P = 0.011). Pain scores were lower in the SP-LC group at 24 and 72 hours postoperatively (both P<0.001). Postoperative CRP and WBC levels were also lower after SP-LC (both P<0.001). Ovarian reserve markers did not differ significantly between groups. The overall complication rate was lower in the SP-LC group (11.59% vs. 25.93%, P = 0.027). Aesthetic satisfaction at discharge was higher after SP-LC (P = 0.006), and PSAS scores were lower at 1 and 3 months postoperatively (P = 0.002 and P = 0.003).

**Conclusion:**

Transumbilical single-port laparoscopic ovarian cystectomy is associated with faster recovery, reduced inflammation, fewer complications, and superior aesthetics without compromising ovarian reserve.

## 1 Introduction

Benign ovarian lesions, including functional cysts, mature cystic teratomas, and endometriomas, are common gynecological conditions affecting women of reproductive age (1, 2). Clinically, these cysts may present with symptoms like pelvic pain, abdominal distension, or menstrual irregularities, and if left untreated, can lead to complications such as torsion or rupture (3, 4). The standard treatment for symptomatic or persistent benign ovarian cysts is currently surgical excision, with laparoscopic ovarian cystectomy being the preferred method due to its minimally invasive nature (5). Although traditional multi-port laparoscopic surgery is effective, it requires multiple abdominal incisions and may be associated with greater postoperative pain and less favorable cosmetic outcomes (6, 7). To address these limitations, single-port laparoscopic cystectomy via the umbilicus (SP-LC) has emerged as a novel minimally invasive alternative. SP-LC uses a single umbilical incision, potentially reducing surgical trauma, minimizing postoperative pain, and enhancing cosmetic satisfaction (8). However, concerns remain regarding surgical complexity, intraoperative cyst rupture, and impact on ovarian reserve (9).

The development of minimally invasive surgery has witnessed the emergence of laparoendoscopic single-site surgery (LESS) and its application in benign gynecological conditions (10). Recent meta-analyses and systematic reviews have indicated that single-port laparoscopy has comparable safety to traditional laparoscopy, with advantages in reducing hospital stay and improving cosmetic outcomes, despite longer operative times (11). Regarding ovarian cystectomy, studies have highlighted the feasibility of the transumbilical approach in reducing intraoperative cyst fluid leakage and postoperative pain, especially for larger cysts with diameters exceeding 8 cm (12). Additionally, advancements in hemostatic techniques, such as the use of oxidized cellulose polymers, have been shown to protect ovarian reserve function during single-port procedures, addressing concerns about potential ovarian damage (13). Despite these promising findings, there is a lack of comprehensive data in the literature comparing SP-LC and MPL concerning inflammatory stress responses, postoperative recovery metrics, and patient-reported aesthetic satisfaction in real-world clinical settings.

Therefore, this retrospective study aims to compare the clinical outcomes and aesthetic satisfaction of transumbilical SP-LC and traditional multiport laparoscopic cystectomy (MPL) in patients with benign ovarian cysts. Innovations include the integration of an objective scar assessment using the Patient Scar Assessment Scale (PSAS) and a focus on the transumbilical approach for large cystic masses. The results of this study aim to provide evidence for optimizing surgical strategies in the management of benign ovarian cysts, emphasizing the potential benefits of minimally invasive approaches on patient recovery, safety, and satisfaction.

## 2 Materials and methods

### 2.1 Study subjects

A retrospective analysis was conducted on 150 patients who underwent laparoscopic ovarian cystectomy at our hospital from October 2023 to September 2025. Inclusion criteria were: (1) preoperative diagnosis of benign ovarian cysts (14); (2) age ≥ 18 years; and (3) complete medical records, including baseline clinical information, perioperative data, laboratory results, postoperative complication records, and follow-up data for aesthetic and scar assessment. Exclusion criteria were: (1) severe coagulation dysfunction; (2) severe heart, lung, liver, or kidney insufficiency; (3) ovarian malignancy; (4) active pelvic or abdominal infection or systemic infection; (5) history of umbilical hernia; (6) history of hormone therapy within the past 6 months; (7) pregnancy or lactation; and (8) presence of mental illness or cognitive impairment.

Based on the surgical technique used, the patient sample was classified into two groups, multi-port (n=81) and single-port (n=69), and included a total of 150 participants. Subjects in the multi-port group received the traditional multi-port laparoscopic ovarian cystectomy, while the subjects in the single-port group received the transumbilical single-port laparoscopic ovarian cystectomy. This study complied with relevant ethical regulations and was approved by the Medical Ethics Committee of West China Second University Hospital, Approval Number (2025–063):.

### 2.2 Surgical technique

All the procedures for both groups were executed by high-volume gynecologic laparoscopic surgeons who had mastered the institutional learning curve for multi-port and transumbilical single-port laparoscopic ovarian cystectomy.

#### 2.2.1 Transumbilical single-port laparoscopic ovarian cystectomy

In the single-port group, patients were placed in a lithotomy position with Trendelenburg tilt and underwent endotracheal intubation for general anesthesia. A vertical incision of 2.5-3.0 cm was made through the umbilicus, and the skin and subcutaneous tissue were incised layer by layer until access to the abdominal cavity was achieved. A single-port multi-channel triport (GelPort, Olympus, Japan) was inserted, and CO2 was insufflated into the abdominal cavity to create pneumoperitoneum, maintaining a pressure of 11–13 mmHg (1 mmHg = 0.133 kPa). A monopolar electrocautery hook was used to make an approximately 1 cm incision in the ovarian cortex, and blunt dissection of the ovarian cortex from the cyst was performed using an aspirator. Bipolar coagulation was employed for hemostasis, and the ovarian cortex was sutured with full-thickness absorbable 3–0 sutures. The ovarian shape was restored, and the specimen was placed in a retrieval bag and removed through the umbilical incision. For cysts larger than 8 cm in diameter, the cyst was carefully brought close to the umbilical incision using a grasper. A purse-string suture with 4–0 silk thread was placed on the cyst surface, and a small incision of approximately 1 cm was made within the purse-string area. The cyst fluid was then aspirated in a controlled manner to reduce cyst volume and minimize fluid spillage. After aspiration, the incision was closed by tightening the purse-string suture. The ovary was then gently exteriorized through the umbilical incision when feasible, and cystectomy was performed under direct visualization, followed by ovarian reconstruction and wound closure. After cystectomy and hemostasis, the ovarian cortex was approximated using full-thickness absorbable sutures to restore the ovarian contour, reduce the residual cavity, and support hemostasis. The reconstructed ovary was then returned to the abdominal cavity, followed by peritoneal lavage and umbilical reconstruction.

#### 2.2.2 Traditional multi-port laparoscopic ovarian cystectomy

In the multi-port group, patients were placed in the lithotomy position with Trendelenburg tilt under general anesthesia. A 10-mm incision was made at the upper margin of the umbilicus, and a 10-mm trocar was inserted as the camera port for placement of a 10-mm 0° laparoscope. Pneumoperitoneum was established using CO_2_ gas with an insufflation pressure of 11–13 mmHg. Visualization of accessory working ports was done laparoscopically. A 5-mm trocar was placed in the left lower abdomen via the reverse McBurney’s point. In contrast, a 10-mm accessory trocar was placed in the left paraumbilical region, approximately 6 cm lateral to the umbilicus. When additional traction or exposure was needed, an additional 5-mm trocar was placed along McBurney’s point in the right lower abdomen. The procedures of cystectomy, hemostasis, and ovarian reconstruction were the same as in the single-port group. At the end of the surgery, the specimen was placed in a retrieval bag and removed via the left paraumbilical 10-mm accessory port.

### 2.3 Observation indicators

#### 2.3.1 Clinical outcomes

##### 2.3.1.1 Perioperative conditions

To assess perioperative conditions, several parameters were noted, including operative time, blood loss, incidence of cyst rupture, first exhaust time, ambulation time, and length of postoperative stay. These indicators compared the aspects of surgical safety and intraoperative trauma, as well as the recovery period at early post-operative stages, for the two analyzed groups.

##### 2.3.1.2 Postoperative pain

Pain was assessed at 2 time points following surgery, 24 and 72 hours. Pain intensity was assessed using the Visual Analog Scale (VAS). The lower the score on the VAS, the better, with a score of 0 designating no pain and a score of 10 indicating the presence of the most severe pain. The VAS has excellent reliability and validity with an intraclass correlation coefficient (ICC) of 0.99 (15).

##### 2.3.1.3 Inflammatory stress response

CRP and WBC levels were measured to assess the inflammatory stress response before and 24 hours after surgical intervention. For this study, CRP levels were quantified using a Cobas e601 automated chemiluminescence immunoassay (Roche, Germany). WBC counts were determined using an automated hematology analyzer (XN-1000, Sysmex, Japan).

##### 2.3.1.4 Ovarian reserve function

Serum estradiol (E2), follicle-stimulating hormone (FSH), luteinizing hormone (LH), and anti-Müllerian hormone (16) levels were determined preoperatively and at 1 and 3 months postoperatively using an automated chemiluminescence immunoassay analyzer (Cobas e601, Roche, Germany).

##### 2.3.1.5 Postoperative complications

Including incision infection, bowel obstruction, pelvic adhesion, and pelvic inflammation.

##### 2.3.1.6 Aesthetic satisfaction

Patients’ satisfaction with the appearance of the incision at discharge was evaluated, including very satisfied, fairly satisfied, neutral, and dissatisfied. Additionally, at 1 and 3 months postoperatively, the Patient Scar Assessment Scale (PSAS) was used to assess patients’ subjective perception of the incision scar. PSAS includes six items: pain, itching, color, hardness, thickness, and irregularity. Each item is scored on a scale of 1 to 10, with 1 indicating normal skin (best) and 10 indicating the worst scar (worst). The total score ranges from 6 to 60, with lower scores indicating better scar condition. The Cronbach’s α coefficient for PSAS is 0.76 (17).

### 2.4 Statistical processing

Statistical analyses were performed using SPSS software (Version 29.0; developed by SPSS Inc., Chicago, IL, USA). A two-tailed P value <.05 was considered statistically significant. The distribution of continuous variables was assessed using the Shapiro-Wilk test combined with visual inspection of histograms and Q-Q plots. Variables that approximately followed a normal distribution are presented as means ± standard deviations (M ± SD). For comparisons of continuous variables between groups, homogeneity of variance was assessed using Levene’s test. When the assumption of equal variance was satisfied, independent samples t-tests were used; when variances were unequal, Welch’s t-test was applied. For continuous variables that did not meet normality assumptions, data were presented as medians with interquartile ranges and compared using the Mann-Whitney U test. Categorical variables are expressed as frequencies and percentages [n (%)] and were compared between groups using the chi-square (χ²) test. A complete-case analysis was performed; patients with incomplete records or missing follow-up data were excluded, and no statistical imputation was applied.

## 3 Results

### 3.1 Basic information

In the comparison of basic information between the multi-port group and the single-port group, no significant differences were observed in age (P = 0.784), BMI (P = 0.589), education level (P = 0.666), marital status (P = 0.746), cyst type (P = 0.993), cyst location (P = 0.858), cyst diameter (P = 0.313), or history of abdominal surgery (P = 0.857) (**Table 1**). These results indicate that the two groups were well-matched in terms of demographic and clinical characteristics, thereby enhancing the comparability of further clinical outcomes between the groups.

**Table 1:** Comparison of basic information between the two groups.

Parameter	Multi-port group (n=81)	Single-port group (n=69)	t/χ^2^	P
Age (years)	35.24 ± 8.13	34.86 ± 8.57	0.274	0.784
BMI (kg/m^2^)	23.06 ± 3.01	22.79 ± 2.94	0.542	0.589
Education level [n (%)]			0.814	0.666
Junior high school or below	18 (22.22%)	12 (17.39%)		
High school	25 (30.86%)	20 (28.99%)		
College or above	38 (46.91%)	37 (53.62%)		
Marital status [n (%)]			0.585	0.746
Unmarried	28 (34.57%)	20 (28.99%)		
Married	48 (59.26%)	45 (65.22%)		
Divorced	5 (6.17%)	4 (5.80%)		
Cyst type [n (%)]			0.014	0.993
Teratoma	43 (53.09%)	37 (53.62%)		
Endometrioma	15 (18.52%)	13 (18.84%)		
Other types	23 (28.40%)	19 (27.54%)		
Cyst location [n (%)]			0.032	0.858
Bilateral	15 (18.52%)	12 (17.39%)		
Unilateral	66 (81.48%)	57 (82.61%)		
Cyst diameter (cm)	5.36 ± 1.51	5.12 ± 1.46	1.012	0.313
Previous abdominal surgery [n (%)]			0.033	0.857
Yes	21 (25.93%)	17 (24.64%)		
No	60 (74.07%)	52 (75.36%)		

### 3.2 Perioperative conditions

For the comparison of perioperative conditions between the multi-port group and the single-port group, cyst rupture incidence and operative time showed no statistically significant differences. Cyst rupture was recorded for the multi-port group in 28 (34.57%) and for the single-port group in 20 (28.99%) cases, with no statistically significant difference found between the two groups (χ²=0.534, P = 0.465) (**Table 2**). Significant differences were recorded for multiple parameters of recovery. Additionally, first exhaust time (P = 0.004), first ambulation time (P = 0.002), and postoperative hospital stay (P = 0.011) were all significantly shorter for patients in the single-port group, suggesting quicker recovery and earlier discharge from hospital. These findings collectively suggest that the single-port procedure may offer benefits in terms of reduced intraoperative blood loss and faster postoperative recovery, potentially leading to improved patient satisfaction and more efficient use of hospital resources.

**Table 2:** Comparison of perioperative conditions between the two groups.

Parameter	Multi-port group (n=81)	Single-port group (n=69)	t/χ2	P
Operative time (min)	84.26 ± 17.94	80.57 ± 16.32	1.308	0.193
Intraoperative blood loss (mL)	48.12 ± 12.85	43.06 ± 11.79	2.496	0.014
Cyst rupture incidence [n(%)]	28 (34.57%)	20 (28.99%)	0.534	0.465
Time to first flatus (h)	18.47 ± 5.74	15.82 ± 5.21	2.937	0.004
First ambulation time (h)	15.63 ± 3.51	13.89 ± 3.17	3.169	0.002
Postoperative hospital stay (d)	4.31 ± 0.92	3.94 ± 0.85	2.577	0.011
Cyst rupture incidence [n (%)]	28 (34.57%)	20 (28.99%)	0.534	0.465

### 3.3 Postoperative pain

**Figure 1:**
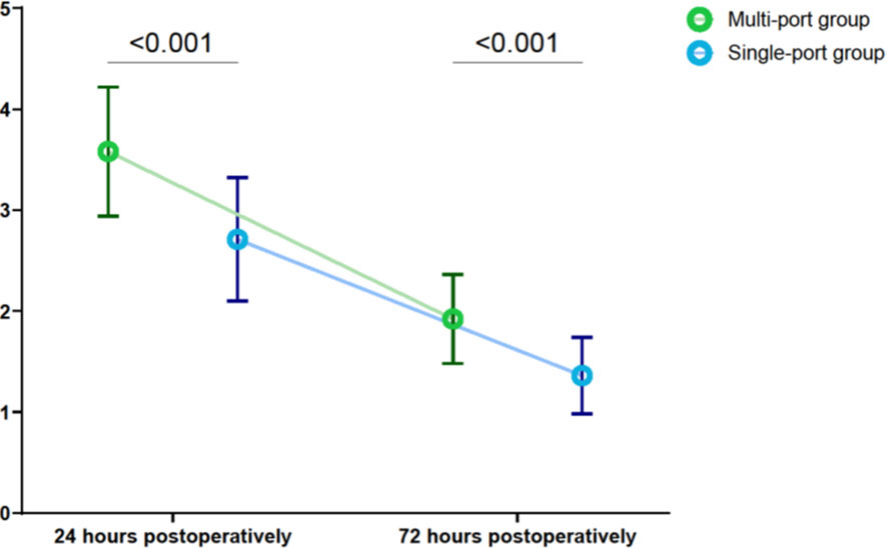
Comparison of VAS scores between two groups (points). VAS, visual analog scale.

In the comparison of VAS scores between the multi-port group and the single-port group, significant differences were observed at both 24 hours postoperatively (P < 0.001) and 72 hours postoperatively (P < 0.001) (**Figure 1**). Patients in the single-port group reported significantly lower VAS scores compared to those in the multi-port group at both time points, indicating that patients experienced less pain following the single-port procedure. This suggests that the single-port approach may be associated with better pain management outcomes in the early postoperative period, potentially contributing to a more comfortable recovery experience for patients.

### 3.4 Inflammatory stress response

In the comparison of inflammatory stress response between the multi-port group and the single-port group, no significant differences were observed in preoperative CRP levels (P = 0.583) or preoperative WBC counts (P = 0.437) (**Table 3**). However, significant differences were noted postoperatively for both parameters. At 24 hours postoperatively, CRP levels were significantly higher in the multi-port group compared to the single-port group (P < 0.001), indicating a more pronounced inflammatory response in patients undergoing the multi-port procedure. Similarly, WBC counts at 24 hours postoperatively were also significantly higher in the multi-port group than in the single-port group (P < 0.001), further supporting the notion of a greater inflammatory stress response associated with the multi-port approach. These results suggest that the single-port procedure may be linked to a milder inflammatory response and lower stress on the body following surgery, which could contribute to quicker recovery times and possibly fewer complications related to inflammation.

**Table 3:** Comparison of inflammatory stress response between the two groups.

Parameter	Multi-port group (n=81)	Single-port group (n=69)	t	P
CRP (mg/L)				
Preoperatively	4.71 ± 0.79	4.78 ± 0.84	0.550	0.583
24 hours postoperatively	26.43 ± 4.05	21.06 ± 3.67	8.445	< 0.001
WBC (×10^9^/L)				
Preoperatively	5.49 ± 1.11	5.35 ± 1.09	0.780	0.437
24 hours postoperatively	8.57 ± 1.63	7.31 ± 1.54	4.825	< 0.001

CRP, C-reactive protein; WBC, white blood cell count.

### 3.5 Ovarian reserve function

In the comparison of ovarian reserve function between the multi-port group and the single-port group, no significant differences were observed across all measured parameters at any time point (**Table 4**). Levels of E2 showed no significant differences preoperatively (P = 0.565), 1 month postoperatively (P = 0.487), or 3 months postoperatively (P = 0.653). Similarly, FSH levels did not differ significantly between groups preoperatively (P = 0.720), 1 month postoperatively (P = 0.539), or 3 months postoperatively (P = 0.723). LH levels also remained comparable preoperatively (P = 0.540), 1 month postoperatively (P = 0.543), and 3 months postoperatively (P = 0.425). Lastly, AMH levels showed no significant differences between the two groups at any assessed time points: preoperatively (P = 0.812), 1 month postoperatively (P = 0.295), and 3 months postoperatively (P = 0.298). This consistency in hormonal profiles implies that patients undergoing either surgical method can expect similar outcomes regarding their ovarian function recovery and preservation.

**Table 4:** Comparison of ovarian reserve function between the two groups.

Parameter	Multi-port group (n=81)	Single-port group (n=69)	t	P
E_2_ (pg/mL)				
Preoperatively	170.23 ± 18.56	168.49 ± 18.12	0.577	0.565
1 month postoperatively	162.15 ± 15.89	160.35 ± 15.44	0.697	0.487
3 months postoperatively	167.91 ± 22.45	166.28 ± 21.73	0.450	0.653
FSH (mU/mL)				
Preoperatively	6.52 ± 1.21	6.59 ± 1.18	0.360	0.720
1 month postoperatively	8.97 ± 1.09	8.86 ± 1.12	0.615	0.539
3 months postoperatively	6.81 ± 1.31	6.73 ± 1.25	0.356	0.723
LH (mU/mL)				
Preoperatively	7.61 ± 1.33	7.74 ± 1.41	0.615	0.540
1 month postoperatively	15.12 ± 1.58	14.96 ± 1.49	0.610	0.543
3 months postoperatively	8.09 ± 1.17	7.94 ± 1.22	0.800	0.425
AMH (ng/mL)				
Preoperatively	3.97 ± 1.21	4.02 ± 1.29	0.238	0.812
1 month postoperatively	3.42 ± 0.95	3.58 ± 0.91	1.050	0.295
3 months postoperatively	3.65 ± 1.09	3.83 ± 0.98	1.045	0.298

E_2_, estradiol; FSH, follicle-stimulating hormone; LH, luteinizing hormone; AMH, anti-Müllerian hormone.

### 3.6 Postoperative complications

In the comparison of postoperative complications incidence between the multi-port group and the single-port group, the overall incidence rate was significantly different (P = 0.027), with the multi-port group exhibiting a higher rate of complications compared to the single-port group (**Table 5**). When examining individual complications, although there were numerical differences in the incidence of incision infection, bowel obstruction, pelvic adhesion, and pelvic inflammation, only the overall incidence rate reached statistical significance. These findings highlight the potential benefits of the single-port technique in reducing the risk of postoperative complications, which could contribute to improved patient recovery and satisfaction.

**Table 5:** Comparison of postoperative complications incidence between the two groups [n(%)].

Parameter	Multi-port group (n=81)	Single-port group (n=69)	χ^2^	P
Overall incidence rate	21 (25.93%)	8 (11.59%)	4.907	0.027
Incision infection	8 (9.88%)	2 (2.90%)		
Bowel obstruction	5 (6.17%)	3 (4.35%)		
Pelvic adhesion	6 (7.41%)	2 (2.90%)		
Pelvic inflammation	2 (2.47%)	1 (1.45%)		

### 3.7 Aesthetic satisfaction

**Figure 2:**
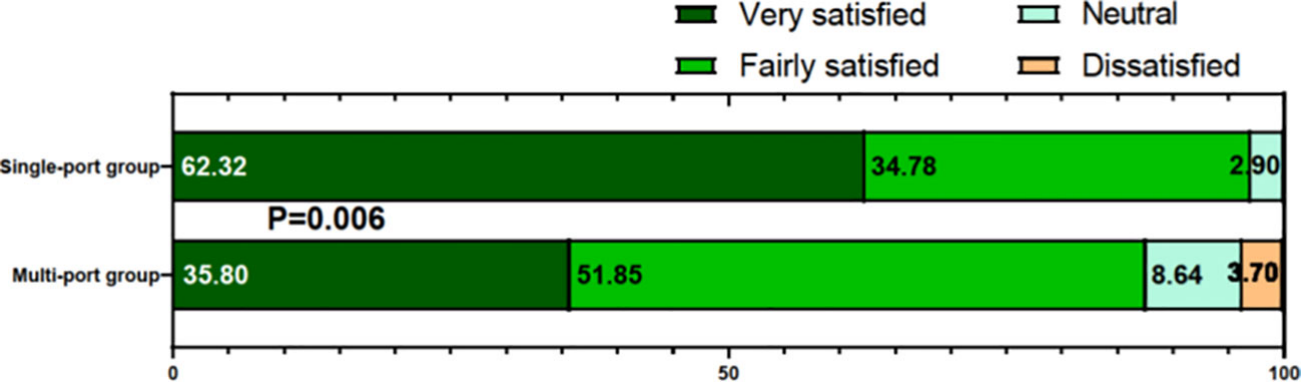
Comparison of incision aesthetic satisfaction between two groups [n (%)].

Regarding the comparison of incision aesthetic satisfaction between the multi-port group and the single-port group, there was a significant difference observed (P = 0.006) (**Figure 2**). Patients in the single-port group reported higher levels of satisfaction with the aesthetics of their surgical incisions compared to those in the multi-port group. A greater proportion of patients in the single-port group expressed being “very satisfied” with the appearance of their incisions. In contrast, more patients in the multi-port group fell into the categories of “fairly satisfied” or “neutral.” Additionally, no patients in the single-port group reported being dissatisfied with the incision aesthetics, in contrast to the multi-port group, where a small percentage of patients did express dissatisfaction. The notable difference in satisfaction levels highlights the potential advantage of the single-port technique in achieving better aesthetic outcomes, which could contribute to improved overall patient satisfaction and psychological well-being postoperatively.

In the comparison of the PSAS scores between the multi-port group and the single-port group, significant differences were observed at both 1 month postoperatively (P = 0.002) and 3 months postoperatively (P = 0.003) (**Table 6**). Patients in the single-port group reported significantly higher satisfaction with their appearance compared to those in the multi-port group at both time points. The consistent difference over the two time periods highlights the sustained benefit of the single-port technique in achieving better aesthetic results, potentially leading to improved overall patient satisfaction.

**Table 6:** Comparison of PSAS scores between two groups (points).

Parameter	Multi-port group (n=81)	Single-port group (n=69)	t	P
1 month postoperatively	18.64 ± 4.21	16.51 ± 3.85	3.203	0.002
3 months postoperatively	15.38 ± 3.96	13.54 ± 3.33	3.056	0.003

PSAS Patient Scar Assessment Scale.

## 4 Discussion

This analysis provides compelling evidence that SP-LC offers distinct clinical and aesthetic advantages over traditional MPL in the treatment of benign ovarian cysts. The findings indicate that SP-LC not only reduces intraoperative trauma and accelerates postoperative recovery but also enhances patients’ aesthetic satisfaction and scar quality. These benefits are achieved without compromising ovarian reserve function, suggesting that SP-LC can be safely incorporated into clinical practice for eligible patients.

The observation of reduced intraoperative blood loss in the SP-LC group aligns with the theoretical advantage of minimal abdominal wall trauma (18). The total length of the umbilical incision is comparable to the sum of the multiport incisions, but involves less dissection through muscle and fascial layers, thereby reducing potential sources of bleeding (19). Additionally, the use of bipolar coagulation and meticulous hemostasis during ovarian reconstruction may have contributed to this finding (20). This finding is consistent with previous meta-analyses, which indicate that single-site procedures generally result in less intraoperative bleeding compared to multiport techniques (21).

Furthermore, the reduction in blood loss may contribute to decreasing the systemic inflammatory response, as evidenced by lower postoperative CRP levels and white blood cell counts in the SP-LC group. CRP is a well-recognized marker of the acute phase reaction to tissue injury, with its elevation correlating to the extent of surgical trauma (22). The observed lower CRP levels suggest that reducing the number of abdominal incisions can mitigate systemic inflammatory cascades, which may impact recovery quality and the risk of inflammation-related complications (23). This attenuated inflammatory response can be attributed to reduced tissue trauma and fewer peritoneal breaches, collectively diminishing the activation of systemic inflammatory pathways (24). Similar reductions in inflammatory markers have been observed in studies comparing single-incision and multi-incision laparoscopic surgeries across different surgical fields, reinforcing the notion that surgical invasiveness is directly linked to postoperative systemic inflammation (25).

The accelerated postoperative recovery indicators in the SP-LC cohort, including earlier first flatus, ambulation, and shorter hospital stays, further confirm the clinical benefits of minimally invasive surgery. The reduction in surgical trauma from single-port laparoscopic surgery may translate to decreased postoperative ileus and earlier restoration of bowel function (26). Early recovery of bowel function and mobility are key indicators of reduced postoperative stress, closely associated with lower morbidity and healthcare costs (27). The observed rapid recovery may be a direct result of reduced inflammatory response and decreased postoperative pain, as evidenced by lower VAS scores at 24 and 72 hours postoperatively. Pain reduction is a consistently reported advantage of single-port procedures, attributed to the absence of multiple separate fascial punctures that can cause neuropathic pain and muscle irritation (28). This is consistent with existing literature, which highlights improved early comfort and reduced analgesic requirements after single-site surgery, thereby enhancing patient satisfaction and facilitating faster mobilization (29).

The overall complication rate in the single-port group was low, mainly due to fewer incision infections and pelvic adhesions. Consolidating surgical wounds into one site simplifies care and reduces infection risks (30). Less peritoneal handling and reduced contact with foreign bodies may explain fewer pelvic adhesions (31). Operative time and cyst rupture rates were similar between groups. Single-port surgery did not prolong operations, indicating effective learning curve management. Similar operational times may partially be due to the skill and experience of the surgeons coupled with the fact that the surgeries were done after the facility had completed its learning curve with respect to single-port laparoscopic surgeries. Similar cyst rupture rates are reassuring, as controlled aspiration via single-port technique can prevent uncontrolled ruptures, supported by previous literature (32).

A key concern with any laparoscopic technique is the potential impact on ovarian reserve function, particularly given the fragility of ovarian tissue and the importance of preserving fertility. Our study shows that SP-LC does not adversely affect postoperative hormone levels. This finding addresses a critical issue with single-port surgery, as increased risk of ovarian damage due to instrument crowding and limited angles could be a concern (33). The results indicate that with appropriate techniques, including careful hemostasis, atraumatic dissection, and precise cortical suturing, the single-port approach can achieve equivalent protection of ovarian function. This outcome is consistent with previous studies demonstrating no difference in ovarian reserve markers between single-incision and multi-incision laparoscopic cystectomy, reinforcing the safety of SP-LC in preserving reproductive potential (34).

Aesthetic satisfaction is a key advantage of single-port technology. At discharge, more single-port patients reported being very satisfied with the appearance of their incisions. The single umbilical incision, hidden within the natural umbilical depression, offers a cosmetic benefit valued by patients, especially young women (35). Fewer visible scars boost overall satisfaction. Previous reviews highlight improved cosmetics as a main advantage of single-port laparoscopy, aligning with patient-centered care principles that consider both clinical efficacy and quality of life (36). It’s important to state that the absolute difference in PSAS scores on a 6–60 point scale, between the two groups, was around two points. This indicates a moderate rather than large clinical magnitude. Despite the single-port group exhibiting lower PSAS scores at the 1- and 3-month post-operative follow-ups, indicating lower perceived scar quality, it is still notable. Therefore, even though the scale comparison was minimal, it can still be described as impactful with regard to patient-perceived aesthetic outcomes. This is especially true considering that the patient population is primarily young adult women, who tend to be more concerned about the quality and appearance of post-operative scarring.

Several limitations are important to discuss even with these positive findings. The retrospective and non-randomized design of this study limits the ability to determine a cause-and-effect relationship and creates a possibility of selection bias. The two groups were similar in demographic data and basic cyst-related variables; however, unmeasured confounding variables, including the experience of the surgeon, the severity of pelvic adhesion, the technical difficulty of each individual case, and the preferences of the patient, may have impacted the surgical technique and the postoperative results. The results may also be less generalizable due to the single-center design of this study. Surgical skill, facility guidelines, and the demographics of the patients all vary from center to center. Although the follow-up period was adequate to study outcomes of an early postoperative assessment and short-term healing of scars, this follow-up period was not adequate to study the long-term outcomes of recurrence and the long-term effects on scar satisfaction. To confirm the findings of this study, more prospective, multicenter, randomized controlled studies should be conducted. The first studies on SP-LC will also need to determine the impact on costs, the impact on resources, the outcomes reported by patients, the time taken to perform a procedure, and the short-term effects due to the widespread use of this surgical technique.

## 5 Conclusion

This retrospective study demonstrates that, compared with traditional multi-port laparoscopic ovarian cystectomy, transumbilical single-port laparoscopic ovarian cystectomy is associated with reduced intraoperative blood loss, accelerated postoperative recovery, attenuated inflammatory stress, lower overall complication rates, and superior aesthetic satisfaction, without compromising ovarian reserve function. These associations support the consideration of single-port surgery as a safe and effective alternative for selected patients with benign ovarian cysts.

## Data Availability

The original contributions presented in the study are included in the article/supplementary material, further inquiries can be directed to the corresponding author/s.
